# A Flexible and Robust Structural Color Film Obtained by Assembly of Surface-Modified Melanin Particles

**DOI:** 10.3390/nano12193338

**Published:** 2022-09-25

**Authors:** Daiki Yoshioka, Keiki Kishikawa, Michinari Kohri

**Affiliations:** Department of Applied Chemistry and Biotechnology, Graduate School of Engineering, Chiba University, 1-33 Yayoi-cho, Inage-ku, Chiba 263-8522, Japan

**Keywords:** photonic materials, structural color, colloidal particles, biomimetics, melanin, polydopamine

## Abstract

In this study, core–shell-hairy-type melanin particles surface modified with a polydopamine shell layer and a polymer brush hairy layer were fabricated and assembled to readily obtain bright structural color films. The hot pressing of freeze-dried samples of melanin particles decorated with a hydrophilic, low glass transition temperature polymer brush results in films that exhibit an angle-dependent structural color due to a highly periodic microstructure, with increased regularity in the arrangement of the particle array due to the fluidity of the particles. Flexible, self-supporting, and easy-to-cut and process structural color films are obtained, and their flexibility and robustness are demonstrated using compression tests. This method of obtaining highly visible structural color films using melanin particles as a single component will have a significant impact on practical materials and applications.

## 1. Introduction

The construction of periodically structured assemblies consisting of particles of uniform size has attracted much attention for its potential applications in various fields, such as electronics, photonics, and plasmonics [1,2,3]. Among them, many studies have reported on the progress for structural color materials obtained by the assembly of uniformly sized colloidal particles because of their high level of expandability and ease of handling [4,5,6]. Structural color films composed of colloidal particles, however, generally have low mechanical strength and require proper curing before they can be used as a material. The immobilization of microstructures using gelation [7,8], sol–gel processes [9,10], and UV curing techniques [11,12], as well as the use of inverse opals with porous structures [13,14], have been reported to be effective in developing materials for practical applications. Furthermore, the use of colloidal particles with polymers grafted onto their surfaces is also useful [15,16]. Ohno et al. [17] reported that structural color films can be easily obtained by hot pressing a mixture of silica and carbon black particles decorated with a polymethyl methacrylate (PMMA) brush at a temperature higher than the glass transition temperature (*T*_g_) of the PMMA brushes [17]. However, in a binary assembly of colloidal particles, differences in the specific gravity and sphericity of the particles often result in uneven coloration [18]. Thus, developing a method to obtain vivid structural color films in the unary assembly of colloidal particles is an important issue.

In general, the whitish structural color obtained via the assembly of colloidal particles is due to multiple scattering. Therefore, improvement of the visibility of the structural color has been investigated by adding light-absorbing reagents such as carbon black [19,20] and graphene [21,22] to the system to appropriately suppress the scattered light. In organisms such as peacocks and *Morpho* butterflies, natural melanin, a biopolymer with excellent light absorption capacity, is used as a component of microstructures to achieve vivid structural coloration [23,24]. Polydopamine (PDA), obtained by the self-oxidative polymerization of dopamine (DA), is known as artificial melanin because its components closely resemble natural melanin [25]. While PDA-based synthetic melanin particles have been used as antioxidants [26] and metal recovery agents [27], we have serendipitously discovered that forming periodic microstructures with uniformly sized melanin particles yields a new type of biomimetic structural color material with excellent visibility [28]. Artificially synthesized melanin particles effectively suppress multiple scattering, resulting in bright structural coloration from periodic structures created by monodisperse melanin particles as a single building block [29]. We have conducted a series of fundamental studies, including the influence of the shape, composition, and assembly conditions of melanin particles on structural coloration [30,31,32,33,34,35] and the visualization of structural color using melanin precursors [36,37].

**Figure 1:**
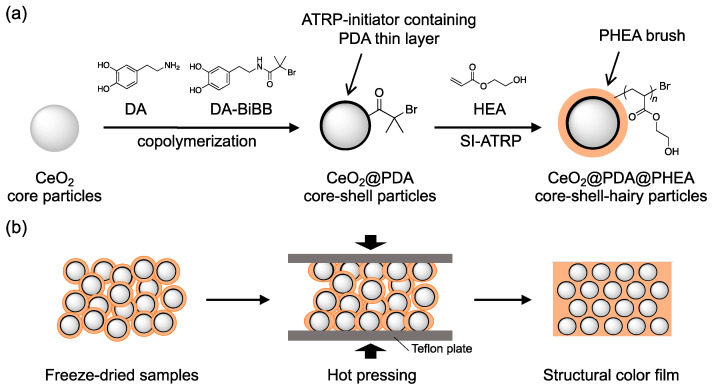
(**a**) Preparation of CeO_2_@PDA core–shell and CeO_2_@PDA@PHEA core–shell-hairy particles. (**b**) Schematic diagram of the preparation of the structural color film by the hot-press method.

In this study, we demonstrated the preparation of flexible and robust structural color films using surface-modified melanin particles with poly hydroxyethyl acrylate (PHEA), which has a low *T*_g_. First, melanin particles with an initiator for atom transfer radical polymerization (ATRP) on their surface were artificially synthesized by copolymerizing dopamine (DA) and ATRP-initiator-modified DA in the presence of cerium oxide (CeO_2_) core particles. The resulting samples were designated CeO_2_@PDA core–shell particles. Next, a hydrophilic PHEA brush layer was formed on the CeO_2_@PDA particles by surface-initiated (SI) ATRP of the HEA monomer. The synthesized samples were designated CeO_2_@PDA@PHEA(X) core–shell-hairy particles (X: the thickness of the PHEA hairy layer (nm)) (Figure 1a). Aqueous dispersions of the obtained particles were dried to prepare solid pellet samples, and the effect of the PHEA hairy thickness on the structural coloration of the pellets was investigated. Finally, freeze-dried samples of CeO_2_@PDA@PHEA particles were hot-pressed and cured to prepare structural color films, and their coloration and physical properties were investigated in detail (Figure 1b).

## 2. Materials and Methods

### 2.1. Materials

Dopamine hydrochloride (DA) was obtained from Sigma-Aldrich Japan Co., LLC. (Tokyo, Japan). Tris(hydroxymethyl)aminomethane (Tris) was obtained from Kanto Chemical Co., Inc. (Tokyo, Japan). Copper(II) bromide (CuBr_2_), L(+)-ascorbic acid sodium salt (NaAsc), *N,N*-dimethylformamide (DMF), and triethylamine (TEA) were obtained from FUJIFILM Wako Pure Chemical Industries Ltd. (Osaka, Japan). 2-Bromoisobutyryl bromide (BiBB), hydroxyethyl acrylate (HEA), and tris [2-(dimethylamino)ethyl]amine (Me_6_TREN) were obtained from Tokyo Chemical Industry (Tokyo, Japan). Deionized water with a resistance of 18.2 MΩ·cm was obtained using a Millipore Simplicity UV system. The CeO_2_ particles coated with polyvinylpyrrolidone (PVP) were provided by Hokko Chemical Industry Co., Ltd. (Tokyo, Japan).

### 2.2. Measurements

Scanning electron microscopy (SEM) micrographs of the samples were obtained using a scanning electron microscope (JSM-6510A; JEOL, Tokyo, Japan). Transmission electron microscopy (TEM) micrographs were obtained using a transmission electron microscope (H-7650; Hitachi, Tokyo, Japan). Reflection spectroscopy was performed using a microscopic spectrophotometer (MSV-370; JASCO, Tokyo, Japan). Angle-changing reflection spectroscopy was obtained using a reflection spectrophotometer (V-650; JASCO, Tokyo, Japan) equipped with a reflection spectroscopy unit (ARSV-732; JASCO, Tokyo, Japan). Photographs of the samples were taken with a digital camera (OM-D E-M10; Olympus, Tokyo, Japan). Infrared absorption spectra were obtained using an attenuated total reflection Fourier transform infrared (ATR–FTIR) spectrometer (FT/IR 4700; JASCO, Tokyo, Japan). The hydrodynamic diameter of the particles in water was measured by dynamic light scattering (DLS) (Zetasizer Nano ZS; Malvern Panalytical, Tokyo, Japan). The thermophysical properties of the polymers were examined using a thermogravimetric analysis (TGA) system (NEXTA-STA; Hitachi, Tokyo, Japan) and a differential scanning calorimetry (DSC) system (DSC7020; Hitachi, Tokyo, Japan). X-ray photoelectron spectroscopy (XPS) measurements were performed using a photoelectron spectrometer (JPS-9030; JEOL, Tokyo, Japan). Freeze drying of samples was conducted using a lyophilizer (FDS-1000; EYELA, Tokyo, Japan). Hot pressing of the samples was performed using a hot-press machine (H300-15; ASONE, Osaka, Japan). Compression tests were performed using a mechanical testing machine (EZ-SX; Shimadzu, Kyoto, Japan). The origin of the compression strain was defined as the point at which the compression stress reaches 0.05 N. Young’s modulus was defined as the slope of the initial linear of the stress–strain curve in the first 5–15% strain range [38].

### 2.3. Preparation of the CeO_2_@PDA Core–Shell Particles

The CeO_2_@PDA core–shell particles were prepared by modifying the method of our previous paper [18,39]. Briefly, a mixture of DA (0.10 g, 0.53 mmol), BiBB (0.10 mL, 0.81 mmol), and TEA (0.11 mL, 0.79 mmol) in DMF (20 mL) was stirred at room temperature for 3 h under nitrogen (N_2_) gas. CeO_2_ core particles (198 nm, 0.20 g), Tris (2.4 g, 20 mmol), and deionized water (180 mL) were added, and the mixture was stirred at r.t. for another 20 h. The resulting samples were separated and purified repeatedly by centrifugation (12,000 rpm for 10 min) and redispersion to obtain CeO_2_@PDA core–shell particles. The diameter of the particles was determined by analysis of the TEM images of 50 particles. The PDA shell layer thickness of the core–shell particles was calculated according to the following formula:

shell layer thickness = [(diameter of core-shell particles) − (diameter of core particles)]/2

### 2.4. Preparation of the CeO_2_@PDA@PHEA(X) Core-Shell-Hairy Particles

HEA (0.31–2.2 mL, 3–21 mmol), CuBr_2_ (20 mg, 0.090 mmol), Me_6_TREN (49 μL, 0.18 mmol) and the CeO_2_@PDA particles (30 mg) dispersed in deionized water (30 mL) were placed in a three-necked flask. The mixture was deoxygenated by purging with N_2_ gas for 15 min. A N_2_-purged aqueous solution of NaAsc (36 mg, 0.18 mmol) was then added to the mixture and stirred at r.t. After 5 h, the polymerization was stopped by purging with oxygen, and the resulting particles were separated and purified repeatedly by centrifugation (12,000 rpm for 10 min) and redispersion, forming CeO_2_@PDA@PHEA(X) core–shell-hairy particles. The diameter of the particles and the hairy layer thickness of the core–shell-hairy particles were determined by the same methods shown above.

### 2.5. Preparation of the Pellet Samples

The pellet samples were obtained by drop casting an aqueous suspension of particles (solid concentration: 10 wt %) onto a silicone rubber plate and drying at room temperature overnight. The average center-to-center distances between the nearest particles (*d*) were determined by 100 pairs of particles obtained from SEM images.

### 2.6. Preparation of Structural Color Films

Solid samples of the CeO_2_@PDA@PHEA(X) particles were prepared by freeze-drying. These samples were sandwiched between two Teflon plates along with a silicone sheet as a spacer and hot-pressed at 50 °C for 15 min to obtain structural color films.

## 3. Results and Discussion

### 3.1. Preparation of CeO_2_@PDA@PHEA Core–Shell-Hairy Particles

**Figure 2:**
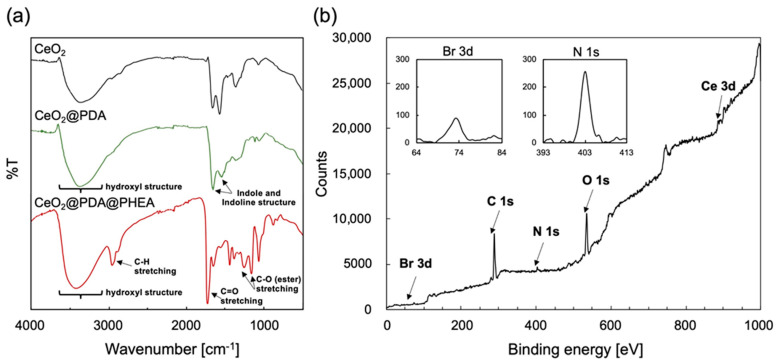
(**a**) IR spectra for the CeO_2_, CeO_2_@PDA, and CeO_2_@PDA@PHEA particles. (**b**) XPS spectrum for the CeO_2_@PDA particles. Insets show the XPS narrow-scan spectra for Br3d (left) and N1s (right).

The CeO_2_@PDA core–shell particles were prepared by the copolymerization of DA and ATRP initiator-bearing DA, i.e., DA-BiBB, in the presence of CeO_2_ core particles. IR measurements of the prepared CeO_2_@PDA particles revealed a broad peak at 3200–3500 cm^−^^1^ due to hydroxyl group structures such as catechol groups, and the characteristic signals were observed at approximately 1500 and 1600 cm^−^^1^ due to indoline and indole structures [40], suggesting the construction of PDA layers on the particle surface (Figure 2a). To evaluate the introduction of BiBB moieties into the PDA shell layer, the XPS spectrum of the CeO_2_@PDA particles was measured. As shown in Figure 2b, in addition to strong C1s, O1s, and N1s signals originating from PDA, weak Br3d signals indicating the presence of BiBB moieties were observed. The Br/N value can be regarded as the BiBB/DA ratio in the PDA shell layer [41], which was determined from the peak areas for the XPS narrow scan spectra for N1s and Br3d to be 0.24 (Figure 2b insets). The volume fraction of the PDA layer within the particles was determined from TGA measurements to be approximately 0.7 wt %. The density of the ATRP initiators on the particle surface, determined by Equation (1) below, was approximately 0.87 molecules nm^−^^2^.

(1) $$\mathrm{Density}\;\left[{\mathrm{molecule}\;\mathrm{nm}}^{-2}\right]=\frac{\frac{\alpha_{\mathrm{organics}}}{\chi }\times N_{A}}{S_{\mathrm{all}}}$$

where *α*_organics_ is the weight of organics estimated from TGA and XPS measurements, *χ* is the molecular weight of the organics, *N*_A_ is the Avogadro constant, and *S*_all_ is the total surface area of the core particles.

**Figure 3:**
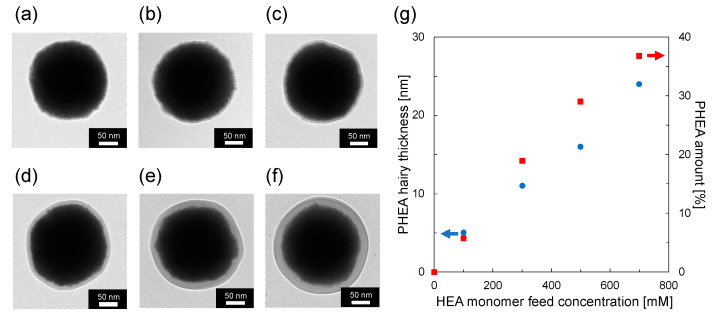
TEM images of (**a**) CeO_2_, (**b**) CeO_2_@PDA, (**c**) CeO_2_@PDA@PHEA(5), (**d**) CeO_2_@PDA@PHEA(11), (**e**) CeO_2_@PDA@PHEA(16), and (**f**) CeO_2_@PDA@PHEA(24) particles. (**g**) Effect of HEA monomer feed concentration on PHEA hairy thickness and PHEA amount in CeO_2_@PDA@PHEA particles.

The CeO_2_@PDA@PHEA core–shell-hairy particles were then prepared by SI-ATRP of HEA in the presence of CeO_2_@PDA particles. The IR spectrum for the CeO_2_@PDA@PHEA particles shows characteristic peaks at 2900 and 1730 cm^−^^1^ due to C-H and C=O stretching vibrations of the PHEA moiety, indicating that the PHEA layers are formed on the particle surface (Figure 2a). Figure 3a–f show TEM images of CeO_2_, CeO_2_@PDA, and CeO_2_@PDA@PHEA(X) particles. The CeO_2_@PDA particles contain a slight shell layer, which is not observed for the CeO_2_ particles. The PDA shell thickness was calculated from the TEM image to be approximately 2 nm. As the HEA monomer feed concentration is increased, a PHEA hairy layer is clearly observed. As shown in the left axis of Figure 3g, the thickness of the PHEA layer, calculated from the TEM image, is controlled from 5 to 24 nm. TEM images of CeO_2_@PDA@PHEA(24) particles measured at low magnification show that all particle surfaces are covered with PHEA hairy layers, demonstrating the usefulness of the present process (Figure S1). Partial coalescence and agglomeration of particles is observed in the TEM images, which may have occurred during the drying of the samples on the TEM grid. To evaluate the presence of aggregation in the obtained particles, the hydrodynamic diameter (*D*_h_) of CeO_2_@PDA@PHEA(X) particles dispersed in water was measured by DLS. As shown in Figure S2, a monodisperse peak with a relatively low polydispersity index (PDI) was observed for all particles, which increases in size without aggregation. Note that the *D*_h_ determined by DLS and the diameter determined by TEM measurements were different because the hydrophilic PHEA hairy chains swelled in water. TGA measurements show that the PHEA amount on the surface of the CeO_2_@PDA@PHEA(X) particles ranges from 5.7 to 36.8 wt %, also indicating controlled PHEA layer building (Figure 3g right axis). DSC measurements for the CeO_2_@PDA@PHEA(24) particles showed a peak at approximately 15 °C, corresponding to the *T*_g_ of PHEA (Figure S3).

### 3.2. Effect of PHEA Hairy Layer Thickness on the Coloration of Pellet Samples

**Figure 4:**
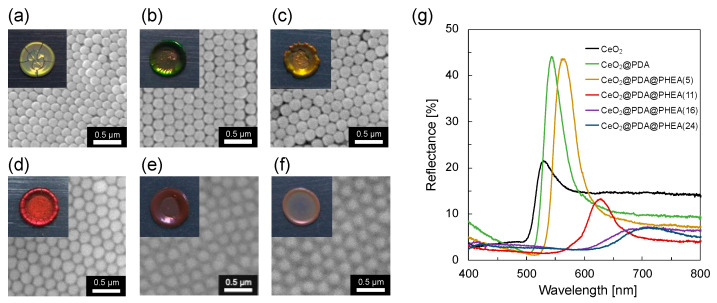
SEM images of pellet samples composed of (**a**) CeO_2_, (**b**) CeO_2_@PDA, (**c**) CeO_2_@PDA@PHEA(5), (**d**) CeO_2_@PDA@PHEA(11), (**e**) CeO_2_@PDA@PHEA(16), and (**f**) CeO_2_@PDA@PHEA(24) particles. The insets show photographs of the obtained pellets. (**g**) Reflection spectra for the pellet samples.

The aqueous dispersion of CeO_2_ particles was milky white, while that of CeO_2_@PDA and CeO_2_@PDA@PHEA(X) particles were both light brown due to the absorption of melanin (Figure S4). Pellet samples were prepared from these aqueous dispersions by drop casting. The green structural color was observed in both pellets prepared from CeO_2_ and CeO_2_@PDA particles (Figure 4a,b, insets). Comparing the reflection spectra for the pellets of CeO_2_ and CeO_2_@PDA particles (Figure 4g), the maximum reflection wavelength of the reflectance spectrum (*λ*_max_) is slightly redshifted due to the increase in particle size due to the PDA shell layer construction. A bright green color is observed for the CeO_2_@PDA pellets, whereas the CeO_2_ pellets are whitish green. The difference in the brightness of the structural color is probably due to absorption by the PDA shell in the CeO_2_@PDA pellet, which suppresses reflections due to multiple scattering in the visible region and emphasizes peaks derived from the structural color [29]. In addition, an increase in the reflection peak intensity is observed. This is probably due to the improved periodicity of the arrangement of the CeO_2_@PDA particles compared to the CeO_2_ particles, as observed from the SEM images shown in Figure 4a,b. The PDA coating fills the surface gaps of the distorted CeO_2_ core particles, resulting in a smooth spherical shape and improving the periodicity of the particle array [18]. When CeO_2_@PDA@PHEA(X) particles are used as components, the color of the pellets shifts from green to red as the interparticle distance affecting the structural color is increased with increasing PHEA hairy layer thickness (Figure 4c–f, insets). As shown in Figure 4g, the *λ*_max_ for the pellet samples is redshifted due to the construction of the PHEA hairy layer. Under practical conditions, Bragg’s law, as expressed by Equation (2), can be applied by considering the effective refractive index (RI) of the system [42]:

(2) $$m\lambda =\sqrt{\frac{8}{3}d^{2}\left(n_{\mathrm{eff}}{}^{2}-sin^{2}\theta \right)}$$

where *m* is the order of diffraction, *λ* is the wavelength of light, *n*_eff_ is the effective RI of the system, *d* is the center-to-center distance between the nearest particles, and *θ* is the angle between the incident light and the sample normal. The SEM images shown in Figure 4c–f indicate that the particles in the pellet samples formed a face-centered cubic (FCC) structure to which Equation (2) can be adapted. The value of *n*_eff_ can be calculated as a weighted sum of the RI of the particles and the gap portion using Equation (3) below [42].

(3) $$n_{\mathrm{eff}}{}^{2}=\sum_{i}n_{i}{}^{2}\Phi_{i}$$

where *n*_i_ and *Φ*_i_ are the RI and volume fraction of their i portion, respectively. For an FCC structure, for the colloidal particles, *Φ* is 0.74. The RIs for the CeO_2_, CeO_2_@PDA, and CeO_2_@PDA@PHEA(X) particles were calculated using the following Equation (4) [43]:

(4) $$n_{\mathrm{particle}}=n_{\mathrm{hairy}}\left[1-{\left(\frac{b}{c}\right)}^{3}\right]+n_{\mathrm{shell}\;}\left[{\left(\frac{b}{c}\right)}^{3}-{\left(\frac{a}{c}\right)}^{3}\right]+n_{\mathrm{core}}\;{\left(\frac{a}{c}\right)}^{3}$$

where *n*_particle_ is the RI of the particles, *n*_hairy_ is the RI of the PHEA hairy layer (1.50 [44]), *n*_shell_ is the RI of the PDA shell layer (1.74 [45]), *n*_core_ is the RI of the CeO_2_ particles coated with PVP, and *a*, *b*, and *c* symbolize the radii of the core, core–shell, and core–shell-hairy particles, respectively. The reflection spectra shown in Figure 4g show that the reflectance for the pellet samples decreases as the thickness of the PHEA hairy layer is increased. In general, the reflectance of light passing from medium 1 to medium 2 is determined by following Fresnel’s equation (Equation (5)) [46]:

(5) $$R={\left(\frac{n_{1}-n_{2}}{n_{1}+n_{2}}\right)}^{2}$$

where *R* is the reflectance of light and *n*_1_ and *n*_2_ are the RIs for medium 1 and medium 2, respectively. Considering the phenomenon that light passing in the air is reflected at the surface of the pellet samples, *n*_1_ can be regarded as the RI of air (1.00) and *n*_2_ can be regarded as the RI of the particles (*n*_particle_). The *n*_particle_ values calculated using Equation (4), the *d* values, the diameter of the particles, and the *λ*_max_ values calculated using Equations (2) and (3) (*θ* = 5°) are summarized in Table 1. The *n*_particle_ is decreased with increasing thickness of the PHEA hairy layer due to the RI of PHEA (1.50). As shown by Equation (5), *R* is a function of the RI of the air and that of the particles. Thus, the reduction in *n*_particles_ may be one of the reasons for the lower reflectance of the pellet samples. The value of *d*, the center-to-center distance between the nearest particles, is smaller than the particle diameter for the CeO_2_ core particles and larger than that for the CeO_2_@PDA@PHEA(24) core–shell-hairy particles, whereas it is nearly equal to the particle diameter for the other particles. It has been reported that the *T*_g_ of PVP decreases below room temperature with increasing water content [47]. Therefore, due to the low *T*_g_ of PVP on the surface of CeO_2_ particles in the aqueous dispersion creating the sample, the CeO_2_ particles can be more densely assembled. For the CeO_2_@PDA@PHEA(24) particles, a large amount of the PHEA hairy layer can result in an expansion of the distance between the particles. Experimental values for *λ*_max_ were compared with calculated values for the component filling the gaps in the particle array: air (RI = 1.00) and PHEA (RI = 1.50) (Table 1). When the CeO_2_, CeO_2_@PDA, and CeO_2_@PDA@PHEA(5) particles are used as components, the experimental values for *λ*_max_ were relatively consistent with the calculated values with air as the gap component. On the other hand, the experimental values of *λ*_max_ for the CeO_2_@PDA@PHEA(11), CeO_2_@PDA@PHEA(16), and CeO_2_@PDA@PHEA(24) particles agree relatively well with the calculated values when PHEA is the gap component, suggesting that the gap component is almost completely filled by the PHEA hairy layer.

### 3.3. Preparation and Characterization of Structural Color Films

**Figure 5:**
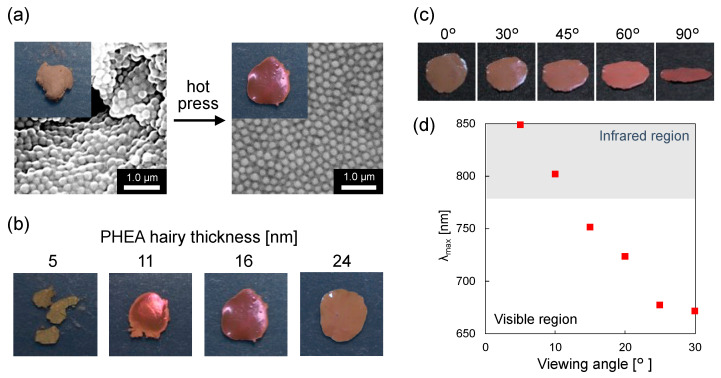
(**a**) SEM images of the freeze-dried samples of CeO_2_@PDA@PHEA(16) before and after hot-pressing. The insets show photographs of the samples. (**b**) Photographs of the structural color films composed of CeO_2_@PDA@PHEA particles with different PHEA hairy thicknesses. (**c**) Photographs and (**d**) *λ*_max_ for the reflection peaks for the structural color film composed of CeO_2_@PDA@PHEA(24) particles viewed from different viewing angles.

Structural color films were prepared by hot pressing the freeze-dried CeO_2_@PDA@PHEA(16) particles. Before hot pressing, the freeze-dried sample shows a brown color due to the light absorption by melanin particles. In contrast, the film obtained after hot pressing at 50 °C shows a bright red color (Figure 5a inset). While no clear reflection spectrum is observed for the sample before hot pressing, the reflection spectrum for the film obtained after hot pressing shows a peak at approximately 750 nm due to the red structural color (Figure S5). This is because the periodicity of the particle array is dramatically improved after hot pressing, as observed from the SEM image shown in Figure 5a. Three films were prepared using CeO_2_@PDA@PHEA(16) particles, and reflection spectra were measured for each film. As shown in Figure S6, the spectral shape and reflectance are almost constant, indicating the reproducibility of the sample preparation using the present method. To investigate the effect of the thickness of the PHEA hairy layer on the formation of the films, structural color films were prepared using CeO_2_@PDA@PHEA(X) particles (Figure 5b). When particles with thin PHEA hairy layer thicknesses (X = 5) are used, no film is found to be formed. On the other hand, particles with thicker PHEA hairy layers (X = 11, 16, and 24) produce structural color films. While the CeO_2_@PDA@PHEA(11) film cracked when force was applied, the CeO_2_@PDA@PHEA(16) and CeO_2_@PDA@PHEA(24) films retained their structure even when bent and showed some flexibility (Figure S7). This indicated that it is desirable to fill the gaps between particles with PHEA hairy layers to form flexible films (vide supra). The angle dependence for the structural color of the film composed of CeO_2_@PDA@PHEA(24) particles was investigated (Figure 5c). While the resulting film viewed from directly above appears brown, which is derived from melanin absorption, the red structural color can be gradually observed as the viewing angle is changed. The reflection spectra for the CeO_2_@PDA@PHEA(24) film measured at different angles shows a decrease in the wavelength of the reflection peak from the infrared region to the visible region (Figure 5d). The film with CeO_2_@PDA@PHEA(24) particles shows an angle-dependent structural color, indicating the formation of a periodic structure for the particles by hot pressing. In the aforementioned structural color pellet fabrication, which is obtained by drop-casting melanin particles dispersed in a solvent, it takes time to obtain pellets because the particles self-assemble as the solvent evaporates. On the other hand, this method demonstrated the simplicity of material preparation, as structural color films were easily obtained in a short time by hot pressing the sample after removing the solvent by freeze-drying.

**Figure 6:**
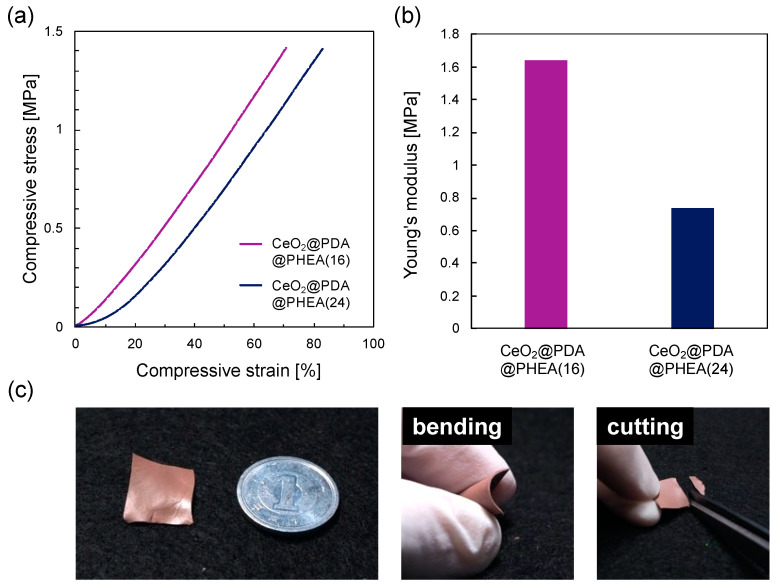
(**a**) Compressive stress as a function of the compressive strain. (**b**) Young’s modulus of the samples obtained from the linear region of the graph shown in (**a**). (**c**) Photographs of the films prepared with the CeO_2_@PDA@PHEA(24) particles when bent or cut.

Compression tests were performed to evaluate the mechanical properties of films consisting of CeO_2_@PDA@PHEA(X) particles. Unfortunately, measurements could not be performed for the CeO_2_@PDA@PHEA(5) and CeO_2_@PDA@PHEA(11) films because the PHEA layer thickness was too thin to maintain the film shape. On the other hand, the CeO_2_@PDA@PHEA(16) and CeO_2_@PDA@PHEA(24) films showed similar stress–strain behavior under the present experimental conditions and did not fracture, regardless of the PHEA hairy layer thickness, suggesting the robustness of the films (Figure 6a). Increasing the PHEA hairy layer decreases Young’s modulus calculated in the 5–15% compressive strain region (Figure 6b), indicating that the film becomes more flexible with increasing the PHEA hairy layer thickness. The appearance of the CeO_2_@PDA@PHEA(16) and CeO_2_@PDA@PHEA(24) films changed little before and after the compression test (Figure S8 insets). Furthermore, their reflection spectra also showed similar shapes, indicating that the structural color of the films was maintained before and after the compression test and the arrangement structure of the particles was preserved (Figure S8). As shown in Figure 6c, the flexible and robust CeO_2_@PDA@PHEA(24) film can be bent and cut.

## 4. Conclusions

We succeeded in the easy preparation of films that show a bright structural color with the surface-modified melanin particles as the single component. Melanin particles with hydrophilic, low *T*_g_ PHEA brushes coated onto a surface can be simply hot-pressed to form structural color films. Hot-pressing freeze-dried CeO_2_@PDA@PHEA particles results in films that exhibit an angle-dependent structural color due to the highly periodic microstructure, with the regularity of the arrangement enhanced by the fluidity of the particles. The resulting structural color film is flexible, self-supporting, and easy to process by cutting. The results from compression tests also confirm the film’s flexibility and robustness. Typical mechanisms for periodic microstructure-based structural color include interference, diffraction gratings, scattering, and photonic crystals [50]. The structural color obtained by the assembly of colloidal particles used in this system can be varied by changing the particle size, refractive index, blackness, and assembly method to achieve various colors [29]. Structural color films have been used in a wide range of applications such as smart displays [51] and strain sensors [52,53] due to their unique coloring properties. The present method can impart an arbitrary polymer layer on the surface of melanin particles, and the resulting particles would be applicable to the fabrication of structural color films with functions such as temperature response [54], mechanochromic [55], and fluorescent properties [56]. Most of these reported materials are prepared by multi-component systems in which light-absorbing materials such as carbon black are added to improve the visibility of the structural color. The method proposed here to obtain highly visible structural color films with melanin particles as a single component will enable simpler material fabrication and expand the possibilities of structural color materials.

## Figures and Tables

**Table 1:** Characterization of pellet samples obtained from the prepared particles.

		CeO_2_	CeO_2_@PDA	CeO_2_@PDA@PHEA(X)			
				X = 5	X = 11	X = 16	X = 24
n_particle_		1.93 *	1.92	1.87	1.81	1.77	1.72
Center-to-center distance (d) (nm)		190 ± 6	204 ± 4	208 ± 4	219 ± 5	236 ± 8	258 ± 17
Diameter of the particles (nm)		198 ± 5	202 ± 7	211 ± 8	223 ± 9	233 ± 7	249 ± 9
λ_max_ [nm]	Experimental values	529	544	565	628	709	714
	Calculated values_gap: air	538	575	572	586	619	660
	Calculated values_gap: PHEA	-	-	604	620	657	702

* The RI of the CeO_2_ particles coated with PVP was calculated to be 1.93 according to Equation (4) above since the thickness of the PVP coating is approximately 10 nm, and the RIs for PVP and CeO_2_ are 1.48 [48] and 2.10 [49], respectively.

## Data Availability

Not applicable.

## Supplementary Materials

The following supporting information can be downloaded at: https://www.mdpi.com/article/10.3390/nano12193338/s1, Figure S1: TEM images of CeO_2_@PDA@PHEA(24) particles measured at low magnification; Figure S2: Hydrodynamic diameter (*D*_h_) of CeO_2_@PDA@PHEA(X) particles dispersed in water as measured by DLS; Figure S3: DSC curve of CeO_2_@PDA@PHEA(24) particles in N_2_ at a 10 °C min^−1^ heating rate. The red arrow indicates the *T*_g_ of the sample; Figure S4: Photographs of aqueous dispersions of CeO_2_, CeO_2_@PDA, and CeO_2_@PHEA(X); Figure S5: Reflection spectra of CeO_2_@PDA@PHEA(16) films before and after hot pressing; Figure S6: Reflection spectra of three separately prepared CeO_2_@PDA@PHEA(16) films; Figure S7: Photographs of CeO_2_@PDA@PHEA(X) films during the bending; Figure S8: Reflectance spectra of CeO_2_@PDA@PHEA(16) and CeO_2_@PDA@PHEA(24) films before and after compression test. The inset shows photographs of the samples before and after the compression test. The center of the films was compressed and the reflectance spectra were measured at the compressed point particles (particle concentration: 1 wt %).
