# Supplementary material for: A Flexible and Robust Structural Color Film Obtained by Assembly of Surface-Modified Melanin Particles
Source: Nanomaterials (Basel). 2022 Sep 25;12(19):3338. doi: 10.3390/nano12193338 (PMC9565605; doi:10.3390/nano12193338)
Supplement: Supplementary file 1 [file nanomaterials-12-03338-s001.zip › nanomaterials-1884881-supplementary.pdf]

nanomaterials

MDPI

Supplementary Materials

A Flexible and Robust Structural Color Film Obtained by Assembly of Surface-Modified Melanin Particles

Daiki Yoshioka, Keiki Kishikawa and Michinari Kohri *

Department of Applied Chemistry and Biotechnology, Graduate School of Engineering, Chiba University, 1-33 Yayoi-cho, Inage-ku, Chiba 263-8522, Japan
* Correspondence: kohri@faculty.chiba-u.jp

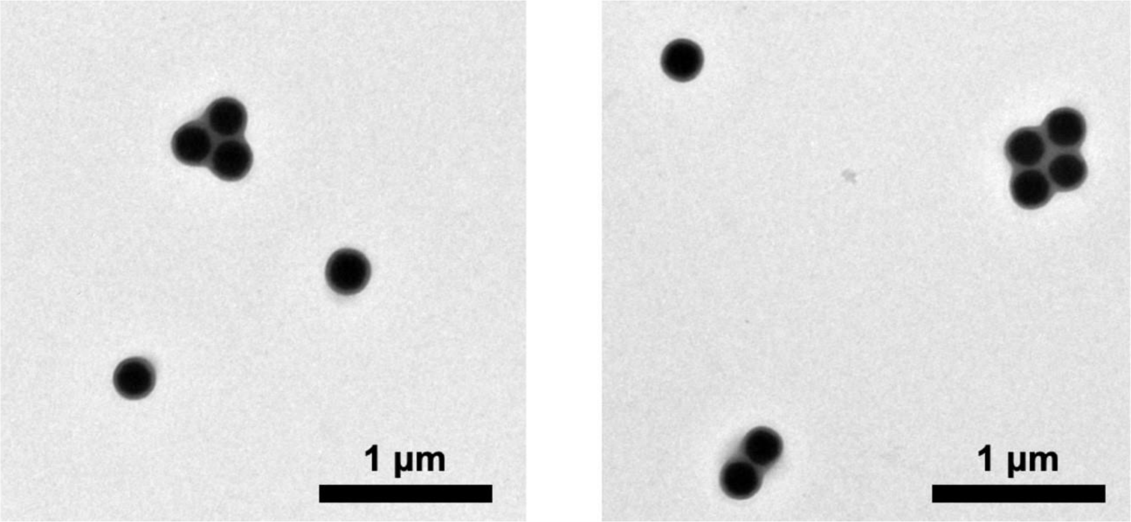

Figure S1. TEM images of CeO_2@PDA@PHEA(24) particles measured at low magnification.

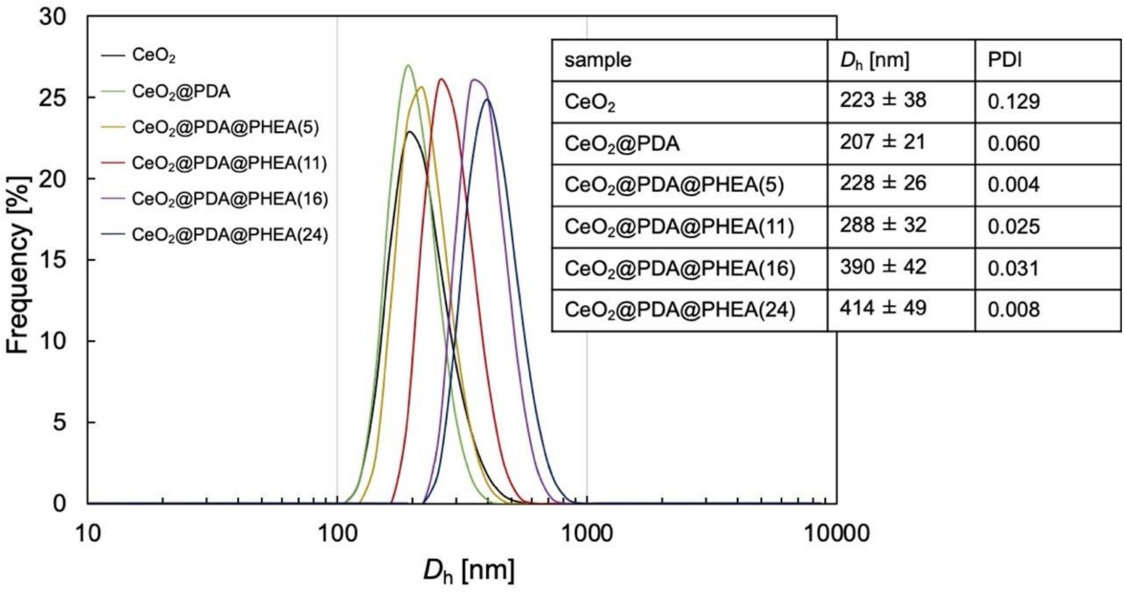

sample	D_h [nm]	PDI
CeO_2	223 ± 38	0.129
CeO_2@PDA	207 ± 21	0.060
CeO_2@PDA@PHEA(5)	228 ± 26	0.004
CeO_2@PDA@PHEA(11)	288 ± 32	0.025
CeO_2@PDA@PHEA(16)	390 ± 42	0.031
CeO_2@PDA@PHEA(24)	414 ± 49	0.008

Figure S2. Hydrodynamic diameter (D_h) of CeO_2@PDA@PHEA(X) particles dispersed in water as measured by DLS.

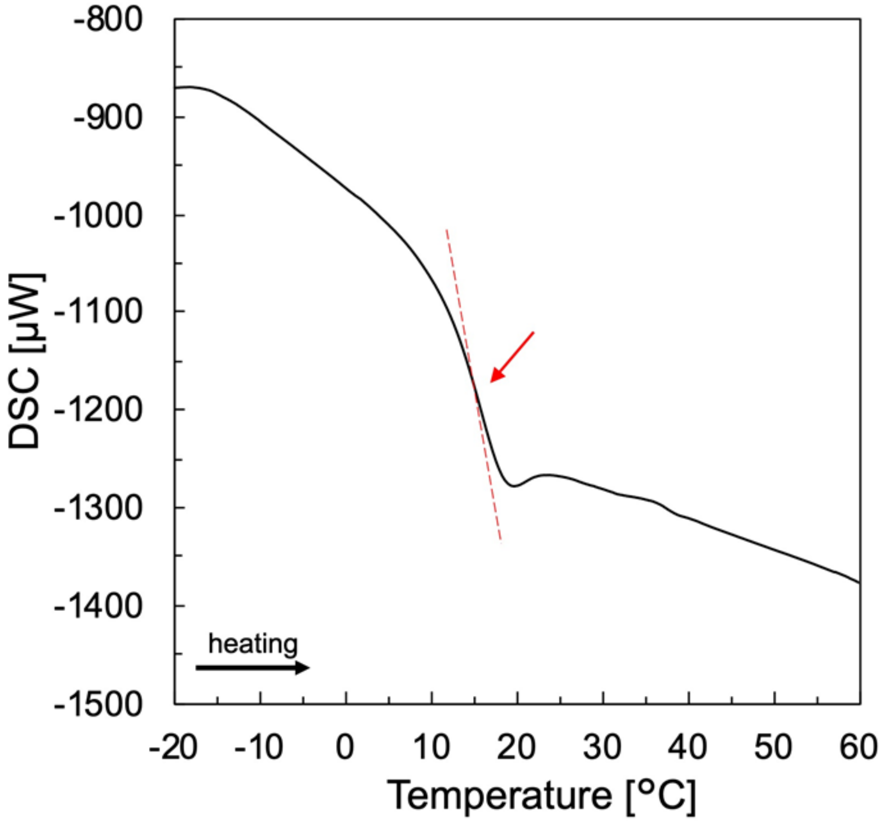

Figure S3. DSC curve of CeO_2@PDA@PHEA(24) particles in N_2 at a 10 °C min^{-1} heating rate. The red arrow indicates the T_g of the sample.

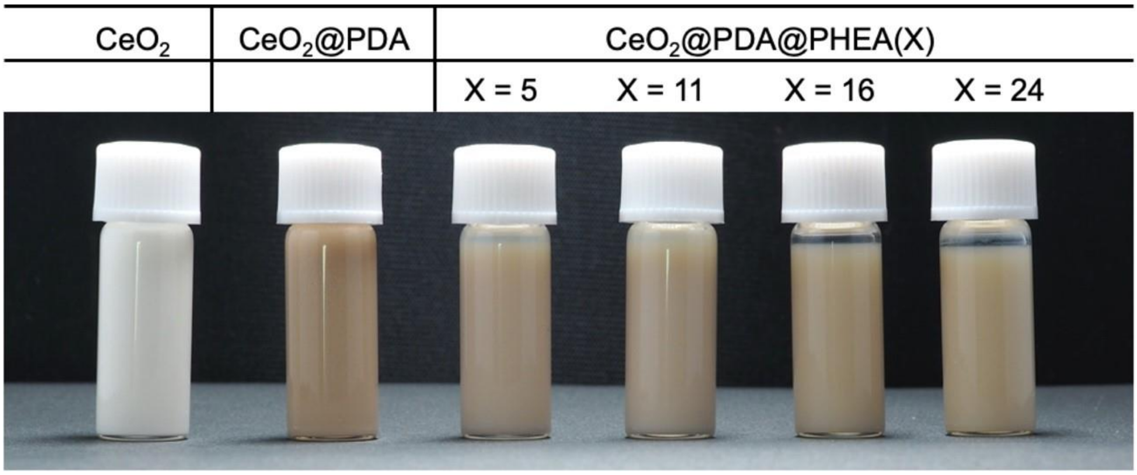

Figure S4. Photographs of aqueous dispersions of CeO_2, CeO_2@PDA, and CeO_2@PHEA(X) particles (particle concentration: 1 wt%).

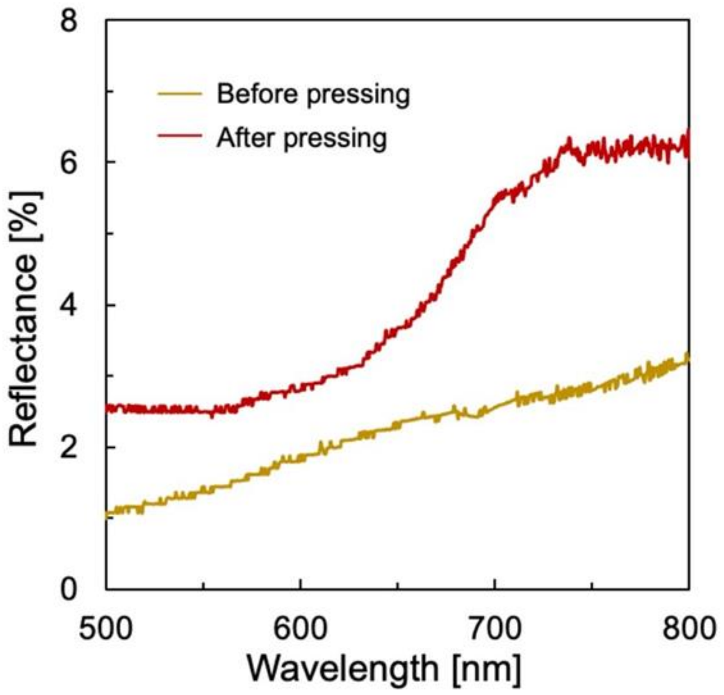

Figure S5. Reflection spectra of CeO_2@PDA@PHEA(16) films before and after hot pressing.

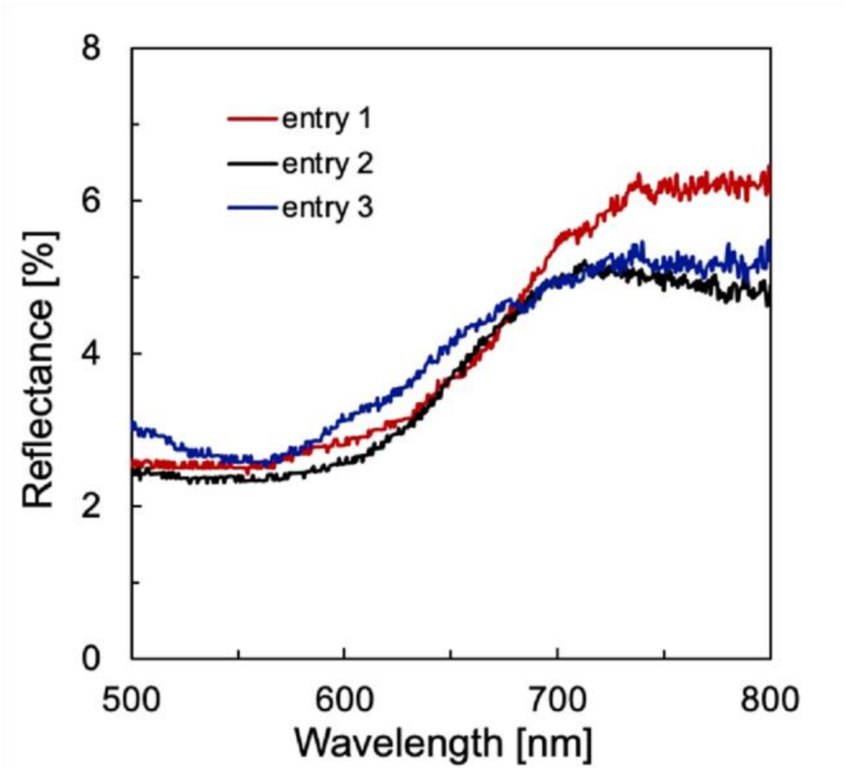

Figure S6. Reflection spectra of three separately prepared CeO_2@PDA@PHEA(16) films.

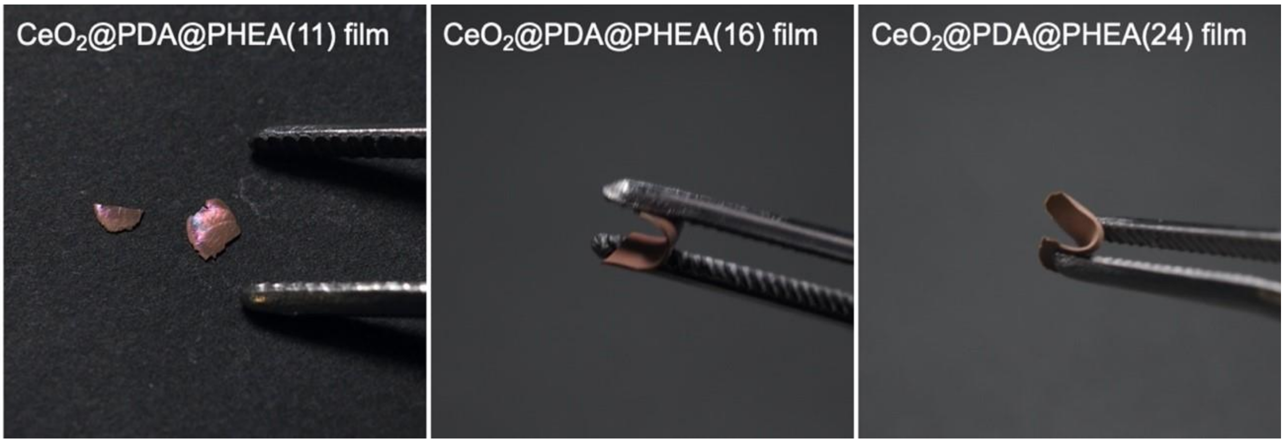

Figure S7. Photographs of CeO_2@PDA@PHEA(X) films during the bending.

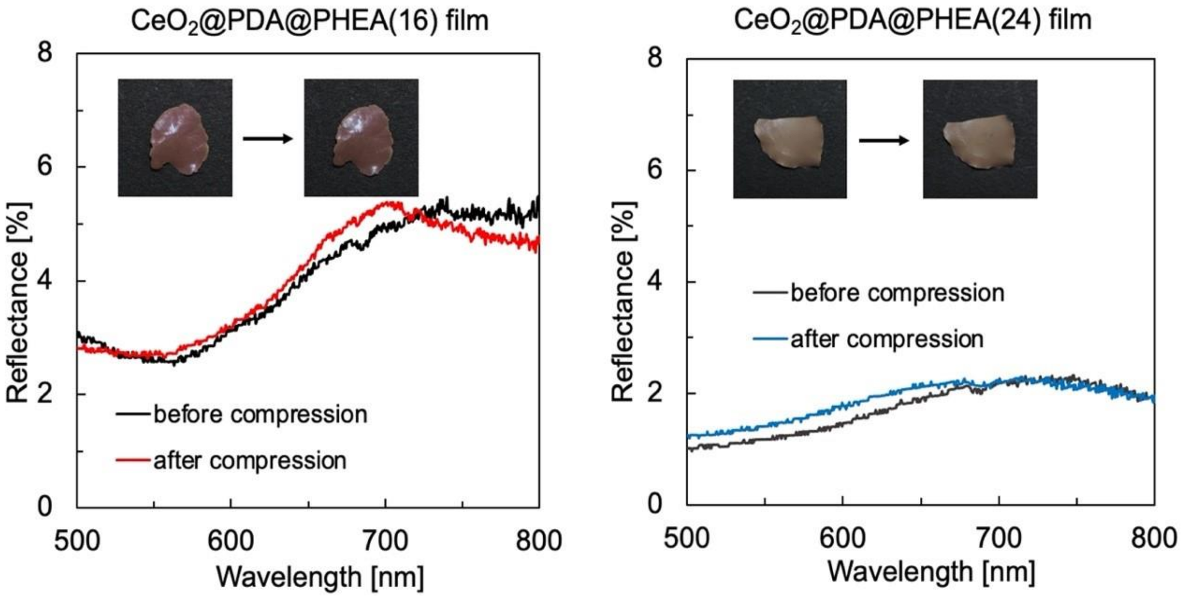

Figure S8. Reflectance spectra of CeO_2@PDA@PHEA(16) and CeO_2@PDA@PHEA(24) films before and after compression test. The inset shows photographs of the samples before and after the compression test. The center of the films was compressed and the reflectance spectra were measured at the compressed point.
